# Heat vulnerability: health impacts of heat on older people in urban and rural areas in Europe

**DOI:** 10.1007/s00508-024-02419-0

**Published:** 2024-08-19

**Authors:** Christina Fastl, Arne Arnberger, Vera Gallistl, Viktoria K. Stein, Thomas E. Dorner

**Affiliations:** 1 Academy for Ageing Research, Haus der Barmherzigkeit, Vienna, Austria; 2 Institute of Landscape Development, Recreation and Conservation Planning, University of Natural Resources and Life Sciences, Vienna, Austria; 3 Division of Gerontology and Health Research, Karl Landsteiner University of Health Sciences, Krems, Austria; 4 Karl-Landsteiner Institute for Health Promotion Research, Sitzenberg-Reidling, Austria; 5 Center for Public Health, Department of Social and Preventive Research, Medical University of Vienna, Vienna, Austria

**Keywords:** Climate change, Hot temperature, Aged, Urban population, Rural ropulation

## Abstract

Exposure to extreme heat is associated with both increased morbidity and mortality, especially in older people. Health burdens associated with heat include heat stroke, diabetes mellitus, hypertension, ischemic heart diseases, heart failure and arrhythmia, pulmonary diseases but also injuries, problems with activities of daily living, and mental disorders. In Europe, there are remarkable spatial differences in heat exposure between urban and less populated areas. In Austria, for example, there is a significant gradual association between population density and the number of heat days, where the gradient of urbanization also follows the gradient of sea level. The European population is continuously ageing, especially in rural areas. Older adults are especially vulnerable to negative health consequences resulting from heat exposure, due to a lack of physiological, social, cognitive, and behavioral resources. Older people living in urban areas are particularly at risk, due to the urban heat island effect, the heat-promoting interplay between conditions typically found in cities, such as a lack of vegetation combined with a high proportion of built-up areas; however, older people living in rural regions often have less infrastructure to cope with extreme heat, such as fewer cooling centers and emergency services. Additionally, older adults still engaged in agricultural or forestry activities may be exposed to high temperatures without adequate protection or hydration. More research is required to examine factors responsible for heat vulnerability in older adults and the interactions and possibilities for increasing resilience in older urban and rural populations to the health consequences of heat.

## Introduction

According to the United Nations Framework Convention on Climate Change (FCCC), climate change refers to changes in weather and temperature patterns over time that can be attributed to human activity [1]. The Intergovernmental Panel on Climate Change (IPCC) defines climate change as any temperature or weather alterations, also including those due to natural variability. Irrespective of the cause, the impacts of climate change can be seen worldwide and across Europe and are projected to increase over time [2].

Changes in weather and temperature related to climate-change have significant implications for human health as they can pose risk factors for a variety of communicable and non-communicable diseases. Changing temperature patterns drive, for example, the expansion of habitats for the hosts of several vector-borne infectious diseases. They also affect food production and thus can contribute to food insecurity and malnutrition. Climate change is accompanied by rising levels of air pollutants, such as small particulates, ozone, fire smoke or allergens, which have detrimental effects for respiratory health. It is currently difficult to estimate the impact that climate change will have on mental health, and health impacts related to societal consequences of climate change. Nevertheless, the evidence for the negative health impacts related to climate change is strong. Probably the highest impact of climate change on health is related to extreme heat [3]. This includes also humid heat waves, which are among the greatest human health threats.

Furthermore, Europe is facing a demographic shift towards rising proportions of persons aged 65 years or older. Projections indicate that by 2050, this age group will make up around 30% of the European population, which is a nearly 10% increase since 2019. In addition, people are getting older [4]. Older adults are especially vulnerable towards the effects of climate change, in particular heat waves [5–9], thus aggravating the public health implications of climate change.

Heat exposure is not constant within countries but differs between urban and less populated areas. Austria, an example of a central European country with a special topography (very flat and very mountainous areas), shows that the spatial distribution of heat is very unequal with higher heat exposure in densely built-up urban areas. While much research has focussed on the heat effects on urban populations, especially on older adults [10–15], distinctive research on heat-related morbidity and mortality for older adults residing in more rural areas is scarce.

This perspective article focuses mainly on one aspect of climate change: heat. The health impacts of the rising temperatures and heat extremes Europe is facing are summarized, focusing on older adults. Furthermore, differences in heat exposure and health outcomes between urban and rural areas are explored. Additionally, potential impacts on research, on society, and on the health burden for Europe, which can be derived from the relationships presented, are discussed.

## Heat in Europe

The temperature in Europe is continuously rising. The mean annual surface temperature was 2.12–2.18 °C higher in the period from 2013 to 2023 than during pre-industrial times (1850–1900), which is nearly twice as high as the global increase. It is projected that the rise in temperature will continue throughout the twenty-first century, albeit the extent of the warming depends on the CO_2_ emission scenario. Northeastern Europe, northern Scandinavia and inner Mediterranean countries are expected to experience the highest temperature increases, while countries from western Europe will likely be less affected [16]. All 10 hottest years on record occurred after 2007, with 2020 and 2023 ranking as the warmest years reported, so far. Heat stress is a measure of thermal comfort and can be approximated via indices, such as the Universal Thermal Climate Index (UTCI), which considers actual temperature, sunshine hours, humidity, heat radiating from surroundings, wind speed, as well as the body’s response to its thermal environment. In 2023, Europe also experienced the highest proportion of “extreme heat stress” days in recorded history, which are defined by a maximum daily UTCI or “feels-like” temperature of 46 °C or higher. The average proportion of days falling into this category across all land areas in Europe was 0.08%, but individual areas in southern European countries reported up to 10 days of extreme heat stress [17].

Heat waves are periods of 3 or more days during which the minimum and maximum temperatures are at unusually high levels for the respective location and thus present a serious health risk. In Europe, the number and severity of heat waves has increased considerably over the past decades. Out of the 50 major heat waves that took place since 1950, 19 occurred between 2012–2021 alone and the most severe heat waves in terms of spatial extent, duration, and degree of temperature anomaly were recorded since 2003 [18]. The minimum temperature indicates whether there is a cool down period overnight [19]. Nights during which the temperature remains above a specific threshold (20 °C for Europe in general, but it can vary depending on the local temperature norms) are also referred to as “tropical nights”. Tropical nights have been on the rise in Europe since the 1980s and are expected to continue to increase in frequency in the future, especially in southern Europe, where, under the worst CO_2_ emission scenario, up to 100 tropical nights annually have been predicted [20].

Temperature extremes differ between urban and rural areas, as evidence from global studies shows. In a study by Menatschi et al. the land surface temperature during warm seasons was compared between urban areas, defined as any built-up area irrespective of city boundaries, and neighboring rural territories, defined as any non-built-up area except of water bodies, within the spatial boundaries of high-density urban centers around the world over 18 years. Urban areas had an average of 2.5 °C higher surface temperatures than non-urban areas. The difference was especially notable during more extreme temperature conditions and there was an increasing temporal trend in the extent of the temperature differences between urban and non-urban areas over the study period [21]. Similarly, in a study by Liu et al., the progression of surface warming in cities around the world was compared to the warming in rural surrounding areas over 20 years and a 29% steeper temperature increase over time was found in cities than in adjacent less populated areas, with more extreme results for larger cities [22]. Thus, temperatures rise at increased paces in urban areas compared to rural areas.

### Example Austria: population density and temperature

The average number of heat days per year, defined as days with a maximum temperature of 30 °C or higher, in the period from 1991 to 2020, measured in 170 different weather stations across Austria, varied from 0 to 27.5 days (mean: 10.0; standard deviation: 7.6 days). Most heat days were recorded in the eastern, flat parts of Austria, in areas where also large cities and urban settlement areas in Austria are located. More than 20 heat days per year were recorded at the 3 measuring stations in Austria’s capital Vienna. The measuring station in the city center of Vienna even reported the second highest values of all Austrian weather stations, with an average of 26.8 heat days per year. Zero heat days per year were only recorded at weather stations in mountainous regions that were at an altitude of at least 1400 m above sea level; however, many heat days were also recorded in some rural areas, mainly in the eastern lowlands of Austria (data derived from [23]).

In Austria, there was a moderate but significant correlation between the mean number of heat days and the population density of the municipality (population per km^2^) in which each weather station is located (Pearson correlation coefficient: 0.359; *P* < 0.001). The population density of the political districts of the respective weather stations also significantly correlated with the number of heat days (Pearson correlation coefficient: 0.320; *P* < 0.001) (own calculations with weather data from [23] and population density data from Wikipedia).

**Fig. 1:**
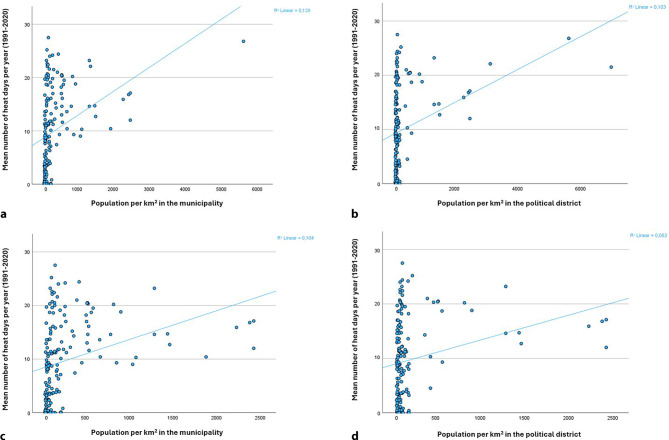
Scatter diagrams: mean number of heat days per year (1991–2020) according to population density (inhabitants/km^2^) in the municipalities (**a** and **c**) and political districts (**b** and **d**), in which the weather stations are located. The weather stations in the capital Vienna are not included in (**c**) and (**d**). Heat days are taken from Geosphere Austria [23], population density was taken from Wikipedia

Figure 1 shows the mean number of heat days by population density in scatter diagrams. There is an approximately gradual relationship between population density (as a proxy for the degree of urbanization) and the number of heat days per year. This connection can be seen both in small areas (municipalities) and in larger areas (political districts). Even if the capital Vienna (the only area with a population density of more than 6000 inhabitants/m^2^) is excluded, this approximately gradual connection is evident. This means that older people in cities, especially those living in densely built-up urban areas without large green and blue spaces, are particularly at risk of exposure to extreme heat [10, 24].

## Health consequences of heat

Extreme heat is associated with a wide range of adverse health outcomes. Hot ambient conditions and heat stress can increase mortality and morbidity, especially the risk of death due to cardiovascular and respiratory issues is elevated [25]. The excess mortality occurs mostly in the first few days of a heat wave, followed by lower than expected mortality. Age, sex, economic and health status/chronic diseases, social isolation, migration background, housing conditions, and residential environment play a role as well as heat wave days during high ozone days [26, 27]. The 2003 and 2022 heat waves were especially severe and resulted in over 70,000 and over 60,000 excess deaths in Europe, respectively, with most fatalities occurring amongst the very old age groups [28–30].

Heat illnesses are best seen as a spectrum of conditions, increasing in severity from heat exhaustion to heat injury and finally to heat stroke. Heat stroke is a life-threatening condition, and the incidence has dramatically increased in the last few decades during heat waves. It is characterized by a high body temperature and central nervous system dysfunction, including delirium, seizures, and coma. Classical heat stroke occurs in immunocompromised individuals, often older sedentary people with chronic diseases during heat waves. The less frequent exertional heat stroke affects young and fit individuals during strenuous activity in hot environments. Long-term effects are believed to stem from systemic inflammation [31].

Non-optimal temperatures were identified as a global death risk by the Global Burden of Disease Study and the rising health impacts of extreme temperatures, including cardiovascular disease, due to climate change and global warming were highlighted by the Lanced Countdown on health and climate change [32]. According to a systematic review, a 1 °C increase in ambient temperature above the optimal temperature threshold for a certain region is associated with an increase in cardiovascular mortality of 3.44% in people aged 65 years or older [8].

Higher temperature increases cardiovascular risk factors such as insulin resistance and diabetes mellitus, worsens blood glucose control in patients with diabetes mellitus and increases blood pressure, especially during the night. These effects along with unhealthy lifestyles, such as low physical activity during heat waves increase the risk for developing cardiovascular diseases. Heat is associated with ischemic heart diseases, heart failure and arrhythmia, and can trigger cardiovascular events such as myocardial infarctions, stroke, or decompensation of heart failure. Pathogenetic factors involved in the development of cardiovascular diseases attributable to heat include peripheral vasodilatation, sweating and evaporation, fluid depletion and electrolyte imbalance, tachycardia, tachyarrhythmia and sympathetic activation, hemoconcentration and hypercoagulability, demand ischemia or atherosclerotic plaque rupture, and thrombosis [32].

Reduced ability to adapt to extreme heat also increases the risk of heat-related injury, partly mediated by problems concentrating during periods of heat and dizziness. Exposure to higher temperatures can also trigger problems with activities of daily living and thereby increase dependency and need of care for older adults. Furthermore, it may negatively affect quality of life and mental health [33].

## Why are older people especially vulnerable to heat?

The higher vulnerability of older adults to heat is due to several intersecting reasons. Many older people do not have the physical, social, and behavioral resources to mitigate the effects of extreme weather events [34, 35]. Older adults are more susceptible especially to heat-related illnesses such as heat stroke, heat exhaustion, and dehydration. Physiologically, ageing diminishes the body’s ability to regulate temperature through processes such as sweating and vasodilation. This impairment is often exacerbated by chronic illnesses common in older populations, such as cardiovascular, respiratory, and renal diseases, which can further hinder thermoregulation and increase the risk of dehydration. Additionally, many older adults take medications that affect their body’s ability to cope with heat, such as diuretics, beta-blockers, and anticholinergics. Older people often are affected by polypharmacy and often take up to 20 different drugs, and it is especially their combination and interaction, which have an unknown effect on people’s vulnerability to heat [36]. Furthermore, social isolation, which also often affects older people, might enhance the health risk associated with heat [35, 37]. The degree of mobility of older adults also contributes to their vulnerability towards heat, especially those who are confined to bed are at risk for heat-related death [38].

## Heat exposure and heat vulnerability among older adults residing in urban and rural areas

There are spatial differences in health threats for older people due to health, especially between urban and rural regions. The “urban heat island” (UHI) effect leads to more pronounced manifestations of heat periods and tropical nights in urban areas than in the surrounding rural regions. This effect is due to the high concentration of buildings, asphalt, concrete and reduced vegetation, which absorb and retain heat. It is particularly apparent in low-income neighborhoods where housing may be poorly insulated, and access to air conditioning is limited. Moreover, urban areas often experience higher levels of air pollution, which can exacerbate respiratory conditions and other health problems, particularly during heat waves. Social factors such as isolation and lack of access to healthcare services also play a significant role in urban settings, where older adults may live alone in apartments with poor ventilation [39, 40]. The extent of the UHI depends on several different factors, most importantly on the percentage of vegetation and blue spaces in the city in question, due to the cooling effect of greenery and water bodies, and the city’s coverage with impervious surface areas as their presence significantly increases the land surface temperature [10, 41]. The seasons also have an impact on UHI intensity, which is typically higher in spring and summer. Other factors include, for example, the size of the city or the population density. UHI is especially pronounced during the nighttime. This is in relation to the thermal capacity of materials found to a greater extent in urban areas, such as asphalt and concrete, as they hinder the cool-down of cities after sunset [42]. In a study from Spain, for example, it was shown that the mean minimum daily temperatures, i.e., the nighttime temperatures, during heat waves were up to 4.1 °C higher in urban than non-urban observatories in all five observed cities. Meanwhile, no comparable differences were found for daytime temperature [43].

Thus, people living in urban areas may be more exposed to extreme heat, especially during the nighttime, when compared to more rural populations and this difference seems to grow over time. Visiting urban blue and green spaces at cooler times of the day can reduce the impact of heat; however, individual health and mobility, availability and accessibility of public green spaces, perceptions of safety and social discrimination because of age or ethnic background experienced in public green spaces may prevent older people from using this heat coping strategy. This can reduce physical activity and lead to increased social isolation. For people with higher economic status, car ownership provides the opportunity to visit cooler regions, and the availability of a second home in rural areas can reduce heat risks [10, 14, 26].

However, older adults residing in more rural territories are also affected by heat and face distinctive challenges in relation to it. Rural areas often have less infrastructure to cope with extreme heat, such as fewer cooling centers and emergency services. Additionally, older individuals in rural settings may have limited access to healthcare facilities and services, which can delay treatment for heat-related illnesses. The physical environment in rural areas, while generally cooler due to more vegetation, can pose significant risks as well, particularly for older adults still engaged in agricultural or forestry activities who may be exposed to high temperatures without adequate protection or hydration. Social isolation in rural areas can also be more pronounced due to geographical distances between parents and children and reduced availability of community health or other support services [44–47].

Additionally, while the number of people living in rural Europe is shrinking, the relative share of people aged 65 years or older is higher in predominantly rural regions than in urban areas for most European countries. Accordingly, also the ratio of older adults to people within working age is higher in most predominantly rural regions in Europe [4]. Thus, a high proportion of the rural population is especially vulnerable towards heat.

## Health impacts of heat on older adults living in urban and rural areas

There is evidence that the increased risk of exposure to extreme heat in urban areas, particularly UHI, also translates into elevated risks of negative health outcomes related to heat, although the extent of this is likely impacted by additional factors, such as housing conditions or socioeconomic status. In a recent analysis of the excess mortality due to heat in 854 European cities from 30 countries over 20 years it was estimated, for example, that around 20,000 deaths per year could be attributed to heat, which amounts to 0.69% of total deaths. The vast majority of excess deaths were estimated within the oldest age group, of 85 years or older [48]. In a study from the US, an overall 1.5% increased risk for cardiovascular disease-related hospitalization for people aged 65 years or older and living in metropolitan areas during periods of extreme heat was found. The risk differed depending on the area and was highest for areas of high UHI intensity, which were responsible for 35% of the total heat-related burden of cardiovascular disease while low UHI areas accounted only for 4% [49].

Distinctive research on heat-related morbidity and mortality for older adults residing in more rural areas is scarce. This may in part be due to the increased risk of misclassifying heat exposure in smaller populations that are spread out across larger areas, or to difficulties in detecting small relative risks due to limited statistical power. Nevertheless, there are some studies that report on and compare heat-mortality risks in both rural and urban areas. In a study from Germany the impacts of heat waves on the mortality of older adults in urban and rural areas were investigated. Elevated mortality risks in both populations, with the highest risk among older people living in the most densely built-up districts of the largest included city were found [50]. In a Swiss study, a significant positive correlation between temperature and all-cause mortality was only reported for people living in urban areas; for rural residents the correlation was smaller and not significant [51]. In a systematic literature review by Odame et al., 11 studies on the associations between heat and mortality among people living in rural areas were identified, mainly from Asian countries, and included in meta-analyses which showed significantly increased risks for both cardiovascular and all-cause mortality for each 1 °C increase in average daily temperature. No indications for notable differences in heat-related mortality risks between urban and rural areas were found [52]. Farmers represent a rural population that is particularly exposed to climate change-related heat. A large part of farm work takes place outdoors, mostly in the warm and hot seasons, often requiring physical exertion despite a variety of mechanical aids, and it is not always possible to postpone the work to cooler days or the off-peak times of the day. In addition, a large part of the farming population is older, especially in family farms, where it is common for family members to continue to do farm work well into old age [47].

Thus, the results of studies that address differences in health outcomes between urban and rural areas paint a mixed picture; however, they underline the fact that both populations face health challenges related to rising temperatures. Table 1 shows the most important differences in the heat vulnerability of older people between urban and rural areas.

**Table 1:** Differences in heat vulnerability in older adults between urban and rural regions

	Urban regions	Rural regions
Sociodemographic factors	Higher population density	Lower population density
	Younger population	Older population
	Often single or small family households	Often multigeneration or large family households
	Smaller social support networks	Bigger social support networks
	Higher levels of education	Lower levels of education
	Higher income	Lower income
	Higher proportion of people with migration background	Lower proportion of people with migration background
	More diverse employment opportunities	Employment concentrated in agriculture and forestry
Topographic factors	Higher density of buildings, asphalt, stone and concrete	More green spaces like forests and agricultural land
	More vertical structures that block wind flow	Less vertical structures which allow ventilation
	Typically in flat warmer regions of the country	Often in mountainous, colder regions of the country
Temperature characteristics	Higher temperature and urban heat islands	Lower temperature
	Lower temperature variation (more tropical nights)	Higher temperature variation (few tropical nights)
	More impervious surfaces	More surfaces like vegetation and soil
Infrastructure	Better access to health care services	Worse access to healthcare services
	Better access to public transportation	Focus on individualized motorised traffic
Airconditioning	Partly common	Less common
Cooling zones	More public cooling zones (e.g. shopping malls, libraries, cultural centers, recreation and senior centres, public pools and splash pads, government buildings)	Less public cooling zones (e.g. churches, senior centers, community halls, natural water bodies)
Challenges in mitigating the health consequences of heat	Increasing green and blue spaces and vertical green Using reflecting building materials Improving urban planning to enhance airflow Removing car traffic	Ensuring access to water, shade and cooling centres Guarantee access to medical care in case of heat strokes and dehydration
Important future research topics	Collecting data of high quality for heat vulnerability, differences in populations, and trends from routine documentation as well as creating new data;	
	Studying spatial differences in urban and rural areas in heat vulnerability, including topographical, infrastructural and socioeconomic conditions;	
	Identifying subpopulations in urban and rural areas regarding heat vulnerability (e.g. those with chronic diseases, living alone, low-income);	
	Modelling and predicting heat vulnerability in the course of climate change in urban and rural regions;	
	Involving the public in both participatory research and projects mitigating health consequences of heat;	
	Translating research findings into effective policies and interventions and thereby overcoming barriers caused by political parties, ideologies and world views	

## Outlook

In summary, temperatures in Europe are continuing to rise and as a consequence, the risk of exposure to extreme heat is increasing for older adults, who are especially vulnerable towards negative health outcomes related to heat and are the fastest growing subpopulation in Europe. Health consequences in older people attributable to heat may be multifaceted and are additionally influenced by a combination of physiological vulnerabilities and socioenvironmental factors. Heat-related health risks, especially cardiovascular morbidity and mortality, are expected to increase in Europe, more in urban than in rural regions, due to climate change. In fact, spatial differences in cardiovascular mortality are already evident in Europe. In the case of Austria, the spatial differences in cardiovascular mortality follows an east-west gradient [53], and thus a similar pattern as the distribution of the frequency of heat days shown in this article. Therefore, it would be interesting to examine how the spatial distribution of cardiovascular morbidity is also driven by the distribution of heat in future research. The combination of high proportions of built-up surfaces and little vegetation in UHIs contributes to especially high temperatures in more densely populated areas, as illustrated by the example of Austria. Nevertheless, heat is also an issue for older adults living in rural regions, where factors such as limited access to cooling facilities or healthcare infrastructure contribute to increased heat vulnerability. Heat vulnerability in rural areas and factors mitigating the health risk in older rural populations in Europe must be better researched in Europe in the future. In addition, awareness of heat impacts among the older rural population should be addressed as well as the heat coping strategies depending on the individual economic and health-related resources and adaptive capacities coupled with socioenvironmental factors. Evidence on whether the spatial differences in heat exposure also translate to differences in heat-associated health outcomes for older adults is mixed and underlines the need for more research in this area as well as into what mitigation options for such outcomes are relevant for which context. Addressing these challenges requires a concerted effort from the scientific and medical communities to implement evidence-based interventions and policies that protect this vulnerable segment of the population from the growing impacts of climate change.
